# The Real-Time Dynamic Monitoring of microRNA Function in Cholangiocarcinoma

**DOI:** 10.1371/journal.pone.0099431

**Published:** 2014-06-11

**Authors:** Xue Chen, Jing Chen, Xinjuan Liu, Zihao Guo, Xiaoxin Sun, Jie Zhang

**Affiliations:** 1 Department of Gastroenterology, Beijing An Zhen Hospital, Capital Medical University, Beijing, China; 2 Department of Gastroenterology, Beijing Tong Ren Hospital, Capital Medical University, Beijing, China; 3 Department of Gastroenterology, Beijing Chao-Yang Hospital, Capital Medical University, Beijing, China; National Institute for Viral Disease Control and Prevention, CDC, China, China

## Abstract

**Background:**

Although many studies have confirmed a relationship between microRNAs (miRNAs) and cholangiocarcinoma (CCA), the real-time dynamics of miRNA function have not been examined.

**Methods:**

miRNA reporter constructs were generated using a recombinant adeno-associated virus vector, which contained complementary sequences for six miRNAs (miR-200a, miR-200b, miR-21, miR-146a, miR-155, and miR-221), along with two independent expression cassettes encoding the fluorescent reporter genes Fluc and Gluc. The spatio-temporal function of each miRNA was monitored both in CCA and control tissues.

**Results:**

All miRNAs participated in CCA development, with distinct patterns of expression over time. The activity of miR-21 was significantly lower in female T3N0M0 CCA tissue relative to controls at three time points, yet was higher in two male T3N1M0 CCA tissues. The difference in miR-200b function between two male T3N1M0 CCA tissues and their corresponding controls peaked at 24 h, while function in a female T3N0M0 CCA was detected only at 72 h. The four remaining miRNAs (miR-200a, miR146a, miR-155, and miR-221) displayed patient-specific activity patterns in both CCA and control tissues.

**Conclusion:**

Significant variability was observed in the temporal function of all six miRNAs, which may play an important role in the development of CCA.

## Introduction

Cholangiocarcinoma (CCA) is one of the most common malignancies derived from bile duct epithelial cells [1]. Due to its slow growth, late metastasis, and lack of effective screening methods, CCA is rarely diagnosed during the early stages of disease when surgical procedures are most effective [2]. One limitation to early diagnosis is a poor understanding of CCA pathogenesis. Histopathological analyses suggest that the presence of primary sclerosing cholangitis, chronic biliary irritation, or choledochal cysts, may predispose individuals to CCA [3]. More recently, studies have identified a role for miRNAs in the development of CCA by altering different cholangiocyte features such as cell cycle, proliferation, migration and apoptosis [4], [5].

miRNAs are endogenous, non-coding RNAs ∼22 nt in length. miRNAs bind complementary sites within the 3′-untranslated (UTR) regions of target messenger RNAs (mRNAs) to control post-transcriptional gene expression, resulting in degradation of target mRNAs, or inhibition of protein translation [6]. These non-coding RNAs are known to regulate cell growth, differentiation, apoptosis, and adhesion, suggesting a direct role for miRNA in the development of multiple cancers, including CCA [7], [8], [9]. MiR21 has been found to be overexpressed in CCA, conferring a variety of oncogenic effects, including inhibition of programmed cell death 4 (PDCD4) and tissue inhibitor of matrix metalloproteinase 3 (MMP3), along with activation of phosphoinositide 3-kinase (PI3K) signaling [10]. miR-200b, a highly overexpressed miR in malignant cholangiocytes, is involved in cell growth, cell dedifferentiation, and oncogenic transformation by targeting the protein tyrosine phosphatase non-receptor type 12, furthermore, miR-200b is believed to contribute to chemoresistance in CCA by modulating the chemotherapy-induced apoptosis [11]. Another miR-200 family member, miR200a, has been shown to up-regulate cytoplasmic and nuclear β-catenin, resulting in enhanced epithelial to mesenchymal transition [12], [13]. Other miRNAs, including miR146 and miR155, are thought to act as mediators of inflammation [14], while miR-221 is an oncogenic microRNA shown to induce tumor angiogenesis in liver cancer [15], [16].

A recent study has found evidence of human biliary extracellular vesicles containing abundant and stable miRNA species [5]. These miRNAs could potentially be optimized into a novel biliary vesicle miRNA-based panel for CCA diagnosis, as well as differential diagnosis for primary sclerosing cholangitis, biliary obstruction, and bile leak syndromes.

Despite recent studies demonstrating a role for miRNAs in CCA pathology, the methodology used was limited. Most miRNA analyses employ a standard approach of cell or tissue lysis and RNA extraction, followed by a variety of complex analyses. These approaches capture only a single time point in an often complex regulatory network, and do not accurately reflect the real-time function of a given miRNA in living cells or organs. Furthermore, the true spatio-temporal role of miRNAs could not be accurately recreated using a simple correlation analysis model, limiting the usefulness of these studies. Recently, we have devised an experiment approach utilizing a recombinant adeno-associated virus (rAAV) vector miRNA sensor named ‘Asensor’. Asensor constructs are constructed by inserting a given miRNA target sequence into the 3′-UTR of a reporter gene containing expression cassettes encoding Gaussia (Gluc) and firefly (Fluc) luciferases in the rAAV [17]. This method allows cellular miRNA function to be monitored continuously and conveniently in real time [18], [19]. In an attempt to study the spatio-temporal physiological feature of miRNAs in CCA, we constructed six Asensor constructs for miR-21, miR-200b, miR-146a, miR-200a, miR-155 and miR-221, and monitored their activity in three CCA and three paired normal specimens.

## Materials and Methods

### Human Tissue Culture

Three distal CCAs and three paired normal specimens were obtained at the time of surgery at Beijing Chaoyang Hospital (Beijing, China). All patients underwent a Whipple’s procedure to remove ampullary lesions. CCAs were diagnosed by endoscopic retrograde cholangiopancreatography (ERCP), magnetic resonance imaging scan, and pathological examination. Both CCA and normal control tissues were classified using the American Joint Committee on Cancer (AJCC) TNM system, along with histopathological examination [20]. All study protocols were approved by the Capital Medical University Ethics Committee (Beijing, China), with all patients providing written informed consent prior to surgery.

Once surgical specimens were removed from patients, samples were immediately divided into tumor and paired normal tissues adjacent to the carcinoma. Tissues (2×2×2 mm in size) were cut and weighed in equal volumes of Dulbecco’s Modified Eagle Medium (DMEM; Invitrogen, Carlsbad, CA, USA), placed into 96-well plates, overlaid with 150-µL DMEM supplemented with 10% fetal bovine serum (Invitrogen, Carlsbad, CA, USA), and 2% penicillin and streptomycin (Invitrogen, Carlsbad, CA, USA) per milligram of tissue. Tissues were then grown in a sterile humidified atmosphere containing 5% CO_2_ at 37°C for at least 3 days.

### miRNA Asensor Construction

miRNA Asensor constructs were generated using AAV vector plasmid pAAV2neo, which contained the complementary sequence of each miRNA (miR-200a, miR-200b, miR-21, miR-146a, miR-155, and miR-221) and two independent expression cassettes encoding Fluc and Gluc, as described in our previous studies. A synthetic poly(A) signal was inserted between the two expression cassettes, which reduced the effects of spurious transcription of the Fluc reporter gene. Gluc was used as a reporter to monitor miRNA activity, while Fluc was used as an internal control to calibrate the transduction efficiency of the specific complimentary miRNA target sequence in the 3′-UTR. The complementary sequences of each miRNA were as follows:

miR-21: (UAGCUUAUCAGACUGAUGUUGA)

miR-200a: (UAACACUGUCUGGUAACGAUGU)

miR-200b: (UAAUACUGCCUGGUAAUGAUGA)

miR-146a: (UGAGAACUGAAUUCCAUGGGUU)

miR-155: (UUAAUGCUAAUCGUGAUAGGGGU)

miR-221: (AGCUACAUUGUCUGCUGGGUUUC).

Control Asensor constructs consisted of an empty viral vector lacking a complimentary miRNA sequence, but were able to express Gluc and Fluc; Asensor controls could not be regulated by miRNAs. Thus, dynamic miRNA activity could be determined using an internal control, an external control, tissue weight, and the Asensor titer calibrated Gluc value. All Asensor constructs were provided by Beijing FivePlus Molecular Medicine Institute (Beijing, China) and Shanghai Biovisualab (Shanghai, China).

### Real-time miRNA Activity Monitoring and Activity

Real-time miRNA activity in living tissue was monitored as described in our previous study [21]. Briefly, preliminary experiments demonstrated that 10^8^ plaque forming units of Asensor per tissue block was the optimal multiplicity of infection for monitoring miRNA activity. Sample blocks were transfected with the appropriate Asensor construct, after which 20-µL medium was collected for Gluc detection at 24, 48, and 72 h. Wells were then supplemented with 20-µL fresh media to ensure the culture system remained unchanged. Tissue blocks were lysed to quantify Fluc (the internal control) 72 h after Asensor transfection.

Gluc and Fluc assay kits were purchased from New England BioLabs (Ipswich, MA, USA) and Promega (Madison, WI, USA), respectively. Both Fluc and Gluc expression were evaluated using a Modulus luminometer (Tuner BioSystems, USA). For the Gluc activity assay, 20-µL medium was added to 50-µL Gluc substrate solution at 24, 48, and 72 h. For Fluc activity, tissue blocks were removed after 72 h, and washed three times with phosphate-buffered saline. Following tissue lysis and centrifugation, 100-µL Fluc substrate solution was added. The levels of Fluc and Gluc activity were quantified using relative light units.

To eliminate accidental and systematic errors, relative inhibiting fold (RIF) was used to monitor miRNA activity. RIF was calculated using the formula [21]:
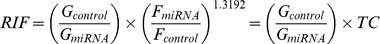



In the formula, G_control_ and G_miRNA_ represent the Gluc activity of the Asensor control and miRNA sensors, respectively, while F_control_ and F_miRNA_ represent the Fluc activity of the Asensor control and miRNA sensors, respectively. TC is the transduction coefficient.

### Statistical Analysis

The SPSS v.17.0 software (Chicago, IL, USA) was used for all statistical analyses. Three cases of CCA and paired normal tissues were compared using repeated measures, wherein *P*<0.05 was considered to indicate statistical significance. Graphs were created using the GraphPad Prism 6.0.1 software (San Diego, CA, USA).

## Results

### Characteristics of Patients

**Figure 1:**
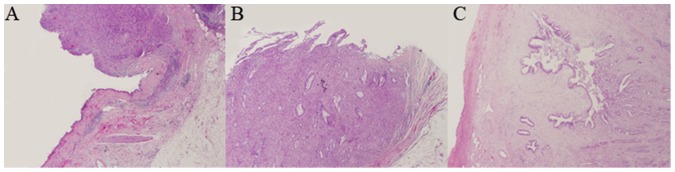
The histological pathology of the three patients. (H&E stain, 40×magnification). CCA: cholangiocarcinoma tissues; N: paired normal tissues adjacent to carcinoma.

CCA and paired normal specimens were obtained from three patients undergoing a Whipple’s procedure at Beijing Chaoyang Hospital, Beijing, China. Basic clinicopathological information of each patient is summarized in Table 1. H&E staining revealed atypical bile duct-like cells extending from the tumor in the normal anatomical location of the bile duct. All tumors exhibited abundant desmoplastic stroma, consistent with a diagnosis of CCA (Fig. 1A–C).

**Table 1:** Clinicopathologic data.

Patient	Age	Gender	Symptom	Location	pathology	Stage	Differentiation
1	64	M	jaundice	E	CCA	T3N1M0	P
2	76	F	pain	E	CCA	T3N0M0	M
3	64	M	pain	E	CCA	T3N1M0	W

Age: patient age at the time of surgery. Gender: male (M) and female (F). Location: extrahepatic (E). Stage: TNM staging. Differentiation: well differentiated (W), moderately differentiated (M), or poorly differentiated (P).

### Validation of Asensor Constructs for Monitoring miRNA Activity

To ensure the feasibility of using Asensor constructs to monitor miRNA activity, a trial run was performed in which we infected patient tissues with a control Asensor construct. Both Gluc and Fluc were highly expressed in both tumor and paired normal controls 24, 48, and 72 h after infection, suggesting that Asensors could be imported into explanted tissues, and survive for at least 3 days in culture. As the expression of Gluc and Fluc varied based upon tissue type and culture conditions, Fluc expression was incorporated as an internal control to measure infection efficiency, tissue specificity, and other factors. Gluc expression will be controlled by each miRNA targeting sequence which was inserted into the 3′ poly A region of Gluc expression cassette.

### Real-time Dynamics of miRNAs in CCA and Control Tissues

To illustrate the real-time dynamics of miRNA function in CCA and control tissues, two CCA-related miRNA Asensor constructs, miR-21 and miR-200b, along with four additional miRNAs, miR-200a, miR-146a, miR-155, and miR-221, were used.

**Figure 2:**
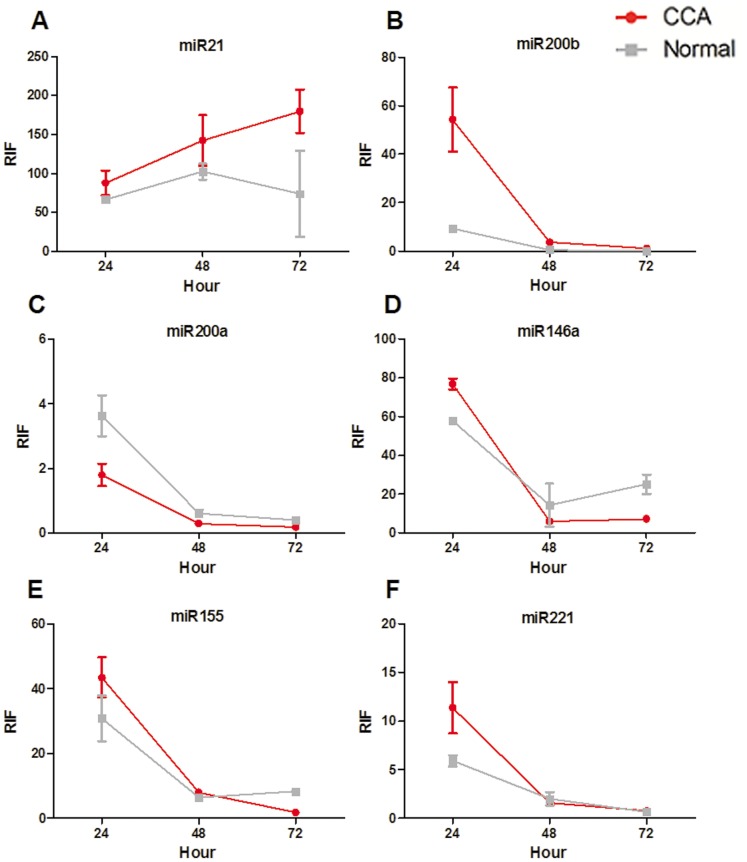
RIF indices (representing activity of each miRNA) of all six miRNAs in patient 1. miR-21, miR-200b, miR-200a, miR-146a, miR-155, and miR-221 activities are shown in A–F, respectively. CCA, cholangiocarcinoma tissues; Normal, paired normal tissues.

For patient 1, a 64-year-old male diagnosed with T3N1M0 CCA, the activity of all miRNAs, except miR-21, weakened from 24 to 72 h in both CCA and control tissues. For miR-21, the RIF indices varied greatly (between 66.43±3.01 and 179.71±27.83), however its activity in CCA tissues increased gradually over time, with miR-21 activity significantly higher than that of controls at both 48 and 72 h (*P*<0.05; Fig. 2A). miR-200b activity was significantly higher in CCA tissues than in controls at all three time points (*P* = 0.0144, 0.0077, and 0.0047, for the 24-, 48-, and 72-h time points, respectively; Fig. 2B); however, this activity decreased steadily over time in both CCA and control tissues.

The lowest degree of variability was seen for miR-200a, with miR-200a activity significantly lower in CCA tissues than in controls at all three time points (Fig. 2C). Average RIFs for CCA tissues were 1.80±0.35, 0.29±0.02, and 0.18±0.02, for the 24-, 48-, and 72-h time points, versus 3.36±0.64, 0.61±0.12, and 0.40±0.04, respectively, for controls. Average RIFs for CCA and control tissues were 76.76±2.68 and 57.76±1.45, 6.02±0.23 and 14.34±11.04, and 7.23±0.23 and 25.12±4.99, at 24, 48, and 72 h, respectively. miR-146a activity was significantly higher in CCA tissues at 24 h, and significantly lower at 72 h, compared to normal controls (Fig. 2D).

For miR-155, the average RIFs of CCA and control tissues at 24, 48, and 72 h were 43.45±6.22 and 30.83±7.13, 7.97±0.49 and 6.37±0.35, and 1.73±0.16 and 8.27±0.58, respectively. Its activity was significantly higher in CCA tissues than in normal controls at 24 and 48 h; however, this difference was lost by 72 h, with miR-155 activity being significantly lower in CCA tissues than in normal controls (Fig. 2E). For miR-221, the average RIFs of CCA and control tissues at 24, 48, and 72 h were 11.37±2.64 and 5.90±0.59, 1.59±0.07 and 1.99±0.67, and 0.76±0.02 and 0.63±0.05, respectively; these differences were statistically significant only at the 24-h time point (Fig. 2F).

**Figure 3:**
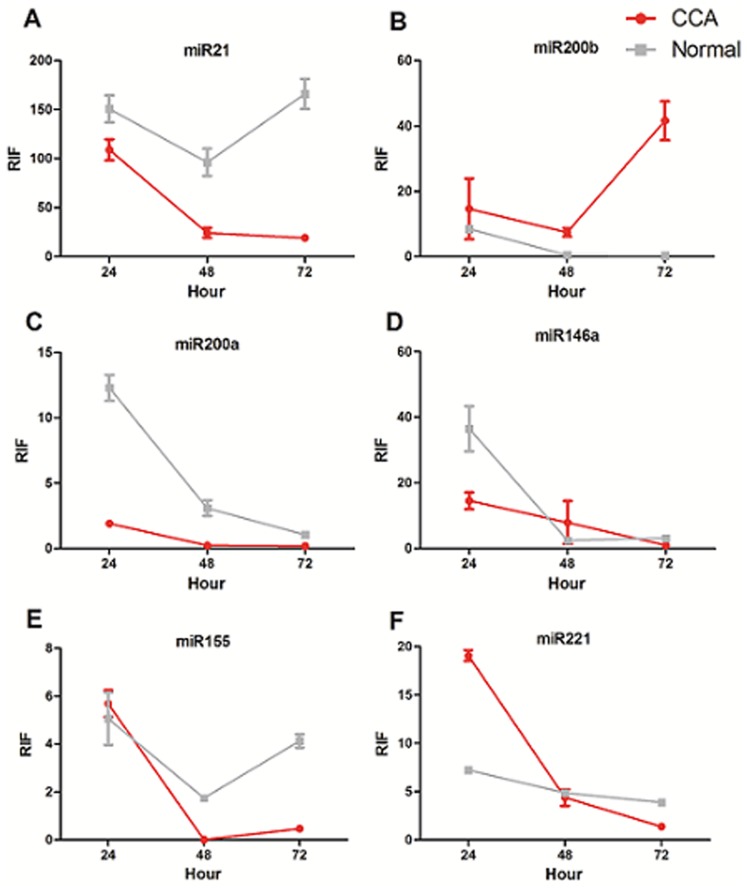
RIF indices of all six miRNAs in patient 2. miR-21, miR-200b, miR-200a, miR-146a, miR-155, and miR-221 activities are shown in A–F, respectively. CCA, cholangiocarcinoma tissues; Normal, paired normal tissues.

Patient 2 was a 76-year-old female diagnosed with T3N0M0 CCA. The RIF indices of miR-21, miR-200b, miR-200a, miR-146a, miR-155 and miR-221 at 24 h in CCA tissues were 108.88±10.82, 14.65±9.26, 1.91±0.18, 14.52±2.61, 5.69±0.56, and 19.07±0.55, respectively, versus 150.71±13.84, 8.51±1.01, 12.30±1.00, 36.46±6.88, 5.06±1.09, and 7.23±0.36, respectively for normal controls. The activities of miR-21, miR-200a, and miR-146a were significantly lower in CCA tissues than in normal controls (*P* = 0.0490, 0.0015, and 0.0280, respectively), while the activity of miR-221 was significantly higher in CCA tissues (*P = *0.0003; Fig. 3).

At 48 h, the RIF indices of miR-21, miR-200b, miR-200a, miR-146a, miR-155 and miR-221 in CCA tissues were 24.31±4.09, 7.43±1.27, 0.23±0.04, 7.87±6.49, 0.01±0.00, and 4.37±0.85, respectively, versus 96.24±13.88, 0.46±0.03, 3.08±0.60, 2.44±0.15, 1.74±0.07, 4.87±0.33, respectively for normal controls. miR-21, miR-200a, and miR-155 were all significantly lower in CCA tissues compared to normal controls (*P* = 0.0029, 0.0064, and 0.0003, respectively), while the activity of miR-220b was significantly higher in CCA tissues (*P = *0.0055; Fig. 3).

By 72 h, the RIF indices of miR-21, miR-200b, miR-200a, miR-146a, miR-155 and miR-221 in CCA tissues were 18.13±2.80, 41.61±5.91, 0.19±0.01, 0.92±0.07, 0.30±0.06, and 1.38±0.08, respectively, versus 18.13±2.80, 0.32±0.02, 1.05±1.13, 3.13±0.14, 4.13±0.28, and 3.88±0.19, respectively, in normal controls. The activities of miR-21, miR-200a, miR-146a, miR-155, and miR-221 were all significantly lower in CCA tissues than in normal controls (*P* = 0.0019, 0.0034, 0.0006, 0.0157, and 0.0012, respectively), while the activity of miR-200b was significantly higher in CCA tissues than in normal controls (*P = *0.0034; Fig. 3).

**Figure 4:**
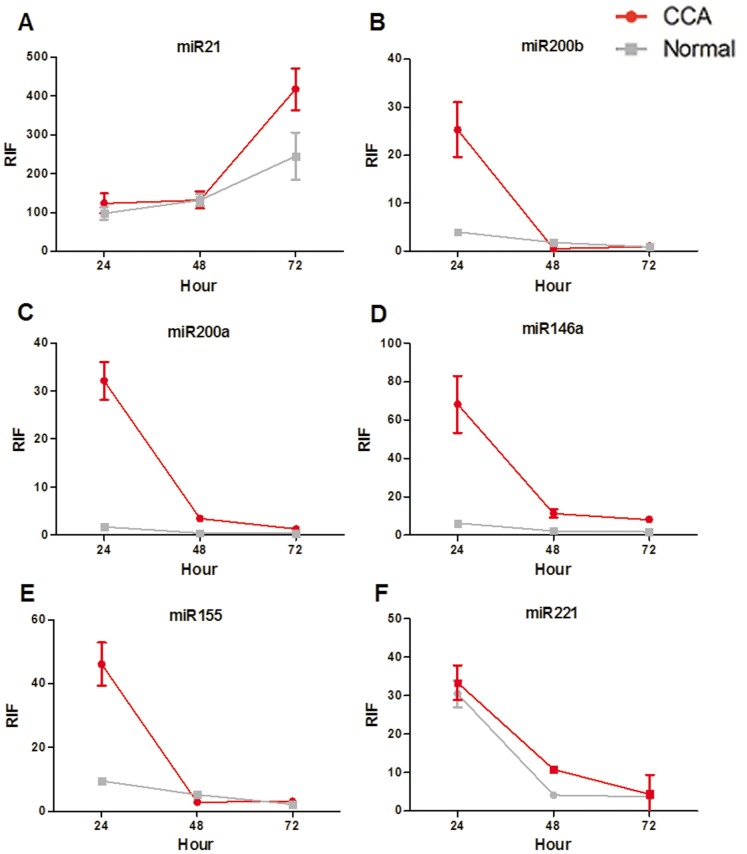
RIF indices of all six miRNAs in patient 3. miR-21, miR-200b, miR-200a, miR-146a, miR-155, and miR-221 activities are shown in A–F, respectively. CCA, cholangiocarcinoma tissues; Normal, paired normal tissues.

For patient 3, a 64-year-old male diagnosed with T3N1M0 CCA, the activities of miR-200b, miR-200a, miR-146a, and miR-155 were significantly higher in CCA tissues than in normal controls at 24 h, with RIF indices of 25.31±5.70 vs. 4.03±0.36 (*P* = 0.0129), 32.14±4.00 vs. 1.75±0.06 (*P* = 0.0028), 68.18±14.82 vs. 6.30±0.45 (*P* = 0.0096), and 46.13±6.74 vs. 9.37±0.56 (*P* = 0.0064), respectively (Fig. 4). At 48 h, the activities of miR-200a, miR-146a, and miR-221 remained significantly higher in CCA tissues than in normal controls, with RIF indices of 3.43±0.68 vs. 0.46±0.01 (*P* = 0.0083), 11.34±2.25 vs. 2.35±0.03 (*P* = 0.0102), and 4.05±0.21 vs. 10.78±0.87 (*P* = 0.0023), respectively (Fig. 4). At 72 h, only miR-21 exhibited significantly higher activation in CCA tissues compared to normal controls, with RIF indices of 417.72±53.50 vs. 244.61±60.95 (*P* = 0.0071; Fig. 4).

## Discussion

Cancer is the result of multiple genetic and molecular mechanisms, with the development of cancer heavily influenced by both the long-term individual physiological microenvironment and individual susceptibility [22]. The processes and mechanisms driving tumor development are therefore likely to change over time with each stage of tumor development. Such a model is best considered using a dynamic, evolutionary-based approach; large-scale studies employing advanced technologies which fail to embrace this line of thinking will invariably fail to fully understand the processes of carcinogenesis. Genome-wide association studies of hepatocellular carcinoma (HCC) have shown that genetic factors vary among HCC niduses [23], with each mechanism corresponding to a specific stage of tumor development. In this study, we profiled six miRNAs via a newly developed miRNA monitoring technology capable of detecting distinct activation differences across multiple time points. This study verifies the aforementioned principles of study design, which can be used to further research in multiple aspects of cancer research.

Current miRNA research tools are dependent primarily on RNA isolation, followed by miRNA discovery, profiling, quantitation, validation, and functional analysis [24], [25]. These classic approaches are not without merit; however, they fail to address the dynamic, time-dependent nature of miRNAs in living tissues. This study offers a new experimental approach involving real-time, dynamic monitoring of miRNAs in live cultured tissues. Although only miR-21 and miR-200a have been directly associated with CCA, a role for all six miRNAs in CCA can be extrapolated from the existing data. miR-21 is highly expressed in CCA, and is associated with occurrence, invasion and metastasis [26], [27], [28], [29]. Overexpression of miR-21 was shown to distinguish malignant from non-malignant bile duct cells with 95% sensitivity and 100% specificity [10]. While these data highlight the key role played by miR-21 in CCA development, the data presented here suggest a more complex picture of miR-21 activity.

miR-21 activity was shown to differ not only between patients, but also between time points. For patient 1, the activity of miR-21 was significantly higher in CCA tissues than in normal controls at all time points, while the opposite pattern was observed in patient 2. This phenomenon could not be accounted for by simple differences in pathophysiologic stages, as patient 3, a 64-year-old male patient with an identical CCA stage of patient 1 (T3N1M0), displayed a third, unique miRNA RIF activity pattern. Further studies are needed to determine the role of patient sex in miR-21 expression.

Similarly, another study reported a strong correlation between miR-200b activity and CCA [11], [30]. The role of miR-200b in the regulation of tumors is related to the epithelial to mesenchymal transition, which can inhibit tumor metastasis [31], [32], [33]. Our study revealed significantly higher miR-200b activity in CCA tissues relative to normal controls at 24 h in patients 1 and 3, with only a small drop off in activity at 48 and 72 h. In patient 2, the activity of miR-200b decreased between 24 and 48 h, but had returned by 72 h. Other miRNAs, including miR-200a, miR146a, miR-155, and miR-221, exhibited specific activities in CCA and control tissues that varied among patients.

We were unable to identify any definitive correlative patterns between the activity of each miRNA and the presence of CCA. While the integrated data overlook many of the details, they suggest that the activities of all six miRNAs are dynamic, and may vary over time, which may better reflective the actual roles of miRNAs in living tissue. To emphasize this point, we have presented our data individually, as opposed to a more integrative analysis. Our experiments utilized live surgical specimens rather than single cells. The microenvironment of explanted tissue samples and cultured cells are often quite different, with tissue blocks more closely replicating the *in vivo* microenvironment [34]. As the effects of miRNAs are highly interdependent [35], [36], the complex *in vivo* molecular network present within explanted tissues may provide a more accurate representation of the miRNA regulation associated with CCA development.

The AAV vector has only mild effects on solid organs, making it a suitable choice for gene therapy and other applications [37]. Aberrant miRNA activity is therefore not a result of AAV infection, as each miRNA construct displayed miRNA- and patient-specific activities. miRNA activity at the 48- and 72-h time points may be influenced by factors associated with transfection, including emergency response, apoptosis, and other unknown factors, however our use of both internal and external control was able to mitigate these effects to some extent. While our approach was not exclude all these confounding factors, miRNA activities *in vivo* are also polytropic, and a major of emphasis of this study. The activity of miRNAs at 24 h post-transfection are likely the best representation of miRNA activity *in vivo*, as the separation time is within 24 h. At this time point, the six-miRNA activity profile displayed significant differences between CCA and control tissues, as well as among patients, which suggests that miRNAs play an active role in CCA development.
